# Thermomechanical Performance Assessment of Sustainable Buildings’ Insulating Materials under Accelerated Ageing Conditions

**DOI:** 10.3390/gels9030241

**Published:** 2023-03-18

**Authors:** Ana Dora Rodrigues Pontinha, Johanna Mäntyneva, Paulo Santos, Luísa Durães

**Affiliations:** 1 University of Coimbra, CIEPQPF, Department of Chemical Engineering, 3004-531 Coimbra, Portugal; 2 Häme University of Applied Sciences, HAMK Tech Research Unit, 13100 Hämeenlinna, Finland; 3 University of Coimbra, ISISE, ARISE, Department of Civil Engineering, 3004-531 Coimbra, Portugal

**Keywords:** thermal insulation materials, performance assessment, ageing, thermal conductivity, Young’s modulus

## Abstract

The reliable characterization of insulation materials in relevant environmental conditions is crucial, since it strongly influences the performance (e.g., thermal) of building elements. In fact, their properties may vary with the moisture content, temperature, ageing degradation, etc. Therefore, in this work, the thermomechanical behaviour of different materials was compared when subjected to accelerated ageing. Insulation materials that use recycled rubber in their composition were studied, along with others for comparison: heat-pressed rubber, rubber_cork composites, aerogel_rubber composite (developed by the authors), silica aerogel, and extruded polystyrene. The ageing cycles comprised dry-heat, humid-heat, and cold conditions as the stages, during cycles of 3 and 6 weeks. The materials’ properties after ageing were compared with the initial values. Aerogel-based materials showed superinsulation behaviour and good flexibility due to their very high porosity and reinforcement with fibres. Extruded polystyrene also had a low thermal conductivity but exhibited permanent deformation under compression. In general, the ageing conditions led to a very slight increase in the thermal conductivity, which vanished after drying of the samples in an oven, and to a decrease in Young’s moduli.

## 1. Introduction

The world crisis in the energy sector allied with the rapid development of national economies makes the environmental degradation a growing serious matter. One major factor that leads to the increasing of greenhouse gas emissions and massive heat losses, which lead to high energy consumption, is the inadequate thermal insulation of buildings. The expenses in heating in the winter and cooling in the summer can be significantly reduced using effective thermal insulation materials, allowing one to cut down the running cost of the annual energy consumption of the air-conditioning systems and heating devices [1,2,3,4,5]. Obviously, these energy savings contribute to lower carbon emissions and overall positive effects on the environment [6].

Independent of the type of structure, the building’s thermal insulation is fundamental to insuring not only its sustainability but the occupant’s comfort [7]. For these purposes, traditional natural materials such as wood [8], rice straw [9], and rice husk [10], have been studied as thermal insulations. Currently, there are several types of thermal insulation materials for buildings [3,4,11]: natural materials, polymer foams and panels, mineral-/ceramic-based materials, aerogels, thermal insulators made from waste materials, composite materials, and metallic or metallised reflective membranes. In the category of the organic materials we can find, for example, cork or extruded polystyrene (XPS), both with low thermal conductivities [3]. The inorganic insulators can comprise mineral wool, calcium silicate, foam glass, perlite, and vermiculate, among others [12].

Catalysed by the consumers’ demand, popular aesthetic factors have gained considerable importance, while, in parallel, the performance consistency of building materials has been known along the years [13]. More recently, the research has shifted to the study of ecological building materials, that have in consideration comfort and public health [14,15]. The utilisation of used tires of rubber in the construction sector is an economical and sustainable solution to achieve the current and upcoming environmental goals, following the campaign “Race to Zero” of the United Nations (UN), that aims to eliminate emissions of greenhouse gases until 2050 [16].

Due to variations in external environmental conditions, such as outdoor temperatures [17,18] and humidity [19,20], the ability of the insulation materials to keep temperatures stable inside buildings also depends on the way these variables affect the thermal conductivity of these materials [21,22]. Several studies report a linear relation between temperature and the thermal conductivity of insulation materials [23,24,25,26,27]. In addition, many studies identify humidity as being extremely impactful on the thermal conductivity of these materials [24,25], especially if the temperatures and humidity values are significantly high.

Studies across several countries about thermal conductivity focused extensively on the effect of variations on humidity and temperature on the intrinsic properties of the insulation materials [28,29]. Other studies used the energy consumption and the wall heat transfer behaviour as metrics to study the thermal conductivity of materials in those buildings [30,31]. The hygrothermal studies of historic buildings and related research projects (e.g., EFFESUS and Hello), have been gaining social strategic relevance and several analyses were also reported [31,32,33,34,35]. Regardless of what is currently known, more can be understood about the effects of the prolonged exposure of insulation materials in actual engineering applications, especially in more extreme cases of temperature and humidity.

In this research project, inspired by environmental issues caused by waste materials, the thermal conductivity of different insulation materials, including some obtained from recycling/reusing of waste, was compared. Considering more extreme variations on the external conditions of buildings, the thermal conductivity values before and after dry-heat, humid-heat, and cold condition cycles were correlated. These included recycled tyre rubber and a composite with rubber and cork. Due to its very low thermal conductivity and good flexibility, a silica aerogel composite with recycled rubber, recently developed by the authors, was also tested along with a commercial aerogel. Moreover, extruded polystyrene (XPS) was characterised as a conventional thermal insulation material. These materials were also characterised regarding their mechanical properties. All materials were subjected to ageing—dry-heat, humid-heat, and cold conditions cycles—during 3 and 6 weeks, and their properties after ageing were compared with the initial values. This assessment, which allows us to obtain conclusions about the best material for different thermal insulation conditions/applications, was not reported previously in the literature.

This article is structured as described next. After this brief introduction, the materials and methods section is presented, where the tested insulation materials are described, as well as the preparation of their test samples. Moreover, it is also described how the structural and thermomechanical characterisation of the samples were performed. To conclude this section, the artificial accelerated ageing cycles equipment and procedures were described. Next, the results and discussion section is presented, starting with the description of the structural and morphological features of the tested insulation materials, followed by the thermal insulation properties, the mechanical performance under compression, and the ageing effect in the materials’ properties and performance. Finally, the main conclusions of this research are presented, including the limitations and future work.

## 2. Materials and Methods

### 2.1. Materials

Five insulating materials that can be used for the insulation of buildings’ constructive elements were selected for this study. They include: recycled rubber strips (MS-R1 from Amorim Cork Composites^®^, Mozelos, Portugal) [36], a rubber_cork composite (MS-R0 from Amorim Cork Composites^®^) [37], aerogel TB strips (aerogel_UK, from SpaceTherm^®^, Blairgowrie, United Kingdom; this aerogel is confined in a plastic coating) [38], extruded polystyrene insulation (XPS, from Topox^®^ Cuber SL, Alpiarça, Portugal) [39] and an aerogel_rubber composite developed by the authors [40]. From the previous materials, the recycled rubber, rubber_cork composite, and aerogel_UK have already been used in previous studies related to the mitigation of thermal bridges effect on lightweight steel-framed (LSF) walls [41]. Moreover, some numerical simulations were performed in a parametric study to assess different setups of an external facade LSF walls [42].

### 2.2. Preparation of Materials for Testing

A total of 128 samples were prepared for the several characterization tests to be performed, as shown in Table 1.

Three types of sample dimensions were used: one for thermal conductivity tests using the Hot Disk transient plane source method, another for thermal conductivity tests performed by the heat flow meter (HFM) method, and the last for the mechanical tests. The samples were cut by a column drill with a skull drill, using different diameters. For the aerogel samples, scissors were used.

For the thermal conductivity tests performed in the Hot Disk equipment, all samples were circular and had a diameter of approximately 40 mm, except for those of aerogel_UK which were square, with the value of each side approximately equal to the diameter above. The thickness of each sample was generally 10 mm. In the case of the rubber_cork composite, two pieces were considered as a sample, since each piece had a thickness of only 5 mm. The thickness of the new aerogel_rubber composite panels was 16 mm. For the thermal conductivity assessment by the HMF method, the samples of the new aerogel_rubber composites were square and had the following dimensions: 215 × 215 mm^2^ and 16 mm in thickness.

For the mechanical tests, all samples were circular and had diameters of approximately 20 mm and 10 mm in height. Once again, in the case of the rubber_cork composite, two stacked pieces were considered, as previously justified.

### 2.3. Structural and Thermomechanical Characterisation of the Samples

The properties of recycled rubber, rubber_cork composite, XPS, aerogel_UK, and the new aerogel_rubber composite were assessed by different characterization techniques.

For the bulk density (*ρ*_b_) evaluation, the weight (measured with a microbalance of 10^−5^ g precision) and the dimensions (measured with a calliper of 0.01 mm resolution) were obtained for regular pieces of the samples.

A Thermal Constants Analyzer TPS 2500 S (Hot Disk, Göteborg, Sweden) was used to measure the thermal conductivity, k This transient plane source method was implemented using the sensor 5501 (diameter of 6.4 mm) between 2 replicates of the same sample at 22 °C. For big panel samples, with square dimensions of 21.5 × 21.5 cm^2^, the thermal conductivity was also determined using the Heat Flow Meter HFM 436/3/1 Lambda, from NETZSCH (Selb, Germany), at 23 °C, following the EN 1946-1:1999 [43].

The samples’ mechanical properties were determined in an Inspekt mini-series equipment (Hegewald & Peschke, Nossen, Germany), performing uniaxial compression-decompression static tests with a strain rate of 1 mm min^−1^, using 1 load cell of 3 kN. Using duplicate samples, two different tests were carried out. To obtain the Young’s modulus and to evaluate the samples’ dimensional stability, the tests were performed using a load cell of 3 kN with a strain from 0 to 25%, with the first property evaluated from the loading stage and the second from the unloading curve. Destructive tests (compression to the limit) were also performed with the same load cell.

### 2.4. Ageing Cycle

To assess the ageing of the studied specimens, an environmental chamber (Espec ARS-0680 of Häme University of Applied Sciences, Hollola, Finland) was used for the cyclic ageing test. The ageing was performed according to the standard ISO 9142. In this type of cyclic ageing, the specimen is exposed for several successive periods under multi-variable conditions on a cyclic basis. The cycle D5 (dry-heat, humid-heat, and cold cycle) was selected. The cycle contained periods of high temperature (55 °C), humid heat (40 °C, >90% relative humidity (RH)) and cold (−20 °C). In total, 1 cycle (168 h) consisted of the following exposure periods: (a) (48 ± 1) h at a temperature of (55 ± 2) °C and at a RH of 10 %; (b) (48 ± 1) h kept at a temperature of (40 ± 2) °C and at a RH of 95 %; (c) (24 ± 1) h kept at a temperature of (−20 ± 3) °C (RH not controlled); and d) (48 ± 1) h kept at a temperature of (40 ± 2) °C and at a RH of 95%.

Before the ageing process, the specimens were conditioned for 24 h at 23 °C and 50% RH. The transition phase to periods a and b was 30 min, and to periods c (−20 °C) and d; the cooling and heating occurred over 1 h. The 1st set of specimens was removed from the chamber after 3 cycles (504 h) and the 2nd set was removed after 6 cycles (1008 h). The thermal conductivity of specimens was measured before and after the ageing.

## 3. Results and Discussion

### 3.1. Structural and Morphological Features

**Figure 1:**
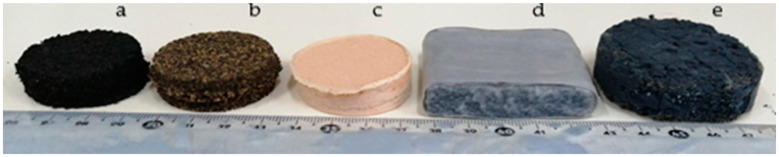
Visual aspect of the different materials, from left to right: (**a**) recycled rubber, (**b**) rubber_cork composite, (**c**) XPS, (**d**) aerogel_UK, and (**e**) new aerogel_rubber composite.

The different tested materials used in thermal insulation of buildings and the one developed by the authors are illustrated in Figure 1.

**Figure 2:**
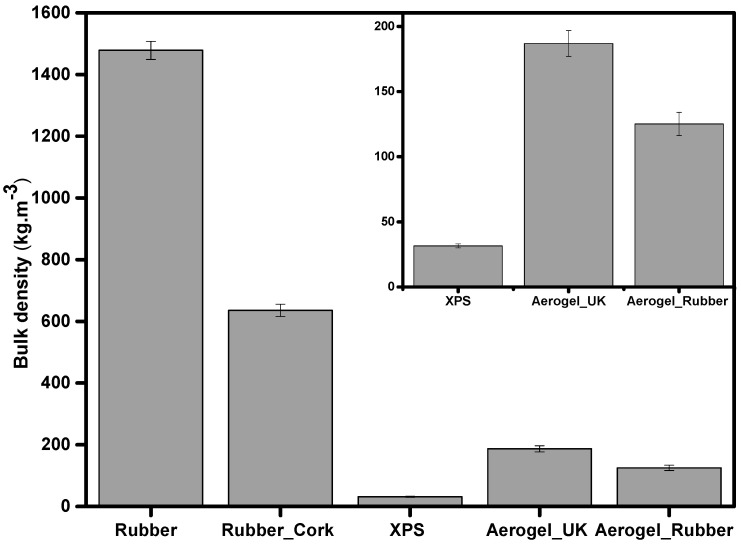
Measured bulk density of the different materials: recycled rubber, rubber_cork composite, XPS, aerogel_UK, and new aerogel_rubber composite.

The results of bulk density for all samples are represented in Figure 2.

Regarding the bulk density values, XPS, aerogel_UK, and new aerogel_rubber composite presented smaller values (31.5 ± 1.5 kg m^−3^, 186.9 ± 9.9 kg m^−3^, and 125.2 ± 8.9 kg m^−3^, respectively) when compared with the rubber and rubber_cork composite. This contributes to better insulation properties. Aerogels have low bulk densities, mainly due to their very high porosity (80–99.8%) [44], with a typical open porous structure composed of silica secondary particles with diameters inferior to 10 nm and pores smaller than 50 nm [45].

Among the tested materials, recycled rubber presents the highest value (1478.3 ± 29.1 kg m^−3^). Cork has a lower density (120–200 kg m^−3^) due to its cellular structure of hollow and closed cells with a small solid fraction [46]. When introducing cork into the composite with recycled tire rubber, the density value decreases (635.3 ± 19.8 kg m^−3^) relatively to the density value of recycled tire rubber, as expected.

### 3.2. Thermal Insulation Properties

**Figure 3:**
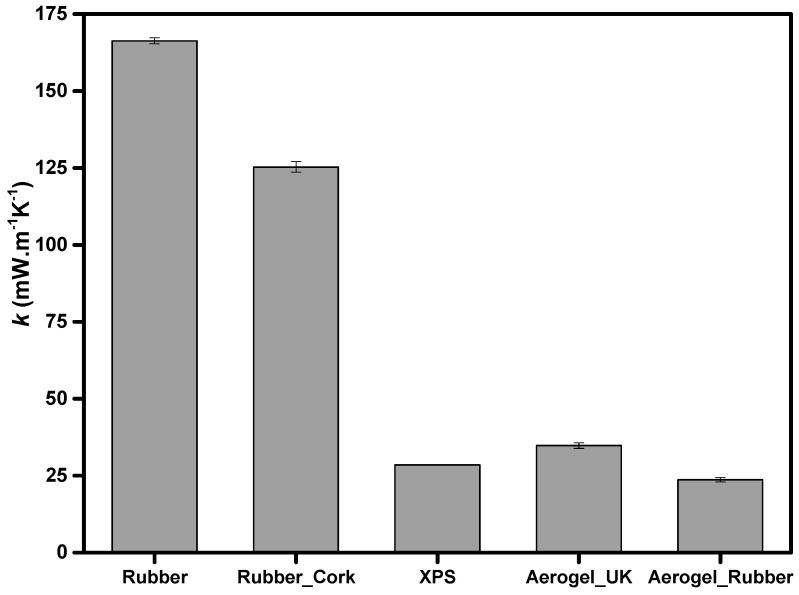
Measured thermal conductivity (k), using the Hot Disk^®^ TPS method, of the different materials at 22 °C: recycled rubber, rubber_cork composite, XPS, aerogel_UK, and new aerogel_rubber composite.

The measurement of the thermal conductivity is crucial because these materials are proposed and used as thermal insulators. The evolution of the thermal conductivity (k), measured by the Hot Disk transient method, follows the trend of bulk density; usually the thermal conductivity and the bulk density values decrease when using aerogels (34.8 ± 0.9 mW·m^−1^·K^−1^ and 23.7 ± 0.7 mW·m^−1^·K^−1^ for the aerogel_UK and the new aerogel_rubber composite, respectively) and XPS (28.5 ± 0.9 mW·m^−1^·K^−1^), as illustrated in Figure 3. The thermal conductivity dependence with the bulk density typically has a U-shape [47,48,49,50,51], with gaseous thermal conductivity having a greater effect for samples with very low densities. The random collisions of gas molecules in aerogels are significantly limited by the nanoporous SiO_2_ skeleton, leading to the Knudsen effect [52]. Like the aerogels, XPS also has many gas-filled pockets which prevent large-scale convection, being good insulators.

The material with the lowest value of thermal conductivity was the new aerogel_rubber composite. When measured by the Hot Disk transient method, it had a thermal conductivity of 23.7 ± 0.7 mW·m^−1^·K^−1^. Thus, the addition of the recycled rubber into the silica colloidal system did not cause an increase in the thermal conductivity of the resulting aerogel material. This is due to the fact that the rubber was dissolved (rubber sol) previous to the addition in the silica sol, as described in earlier work [40]. Therefore, it is molecularly mixed with the silica structure. In the other samples, the presence of solid rubber increased the value of thermal conductivity, with the highest value for the rubber strip samples.

The results of the Hot Disk sensor 5501 are representative of the thermal conductivity of these samples, since it is assessed considering a large volume for the heat transfer. It is expected that for larger samples, with size compatible with Heat Flow Meter measurements (negligible boundary effect), the thermal conductivity may decrease even further. This was verified, as in the case of the thermal conductivity measured by the HFM for the new aerogel_rubber composite the thermal conductivity reached a value of 16.4 ± 1.0 mW·m^−1^·K^−1^.

### 3.3. Mechanical Performance under Compression

**Figure 4:**
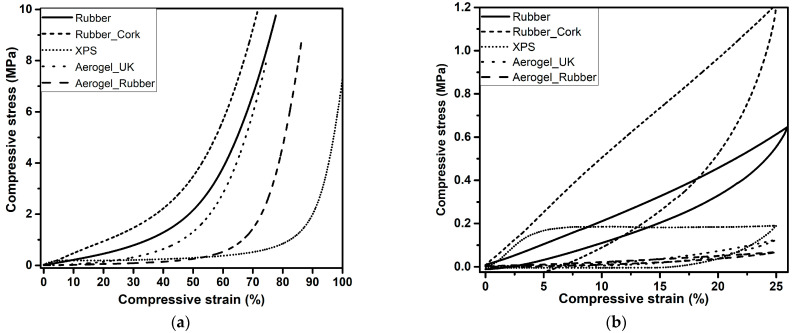
Mechanical behaviour of the rubber, rubber_cork composite, XPS, aerogel_UK, and new aerogel_rubber composite. (**a**) Uniaxial compression with a load cell of 3 kN, and (**b**) reversible compressive stress-strain curves of the composites until 25% strain.

The curves obtained from the destructive tests for all samples are represented in Figure 4a). Despite all the samples being tested under the same conditions, up to the maximum load of the compression cell (3 kN), different values of the maximum compressive strain were obtained for the tested materials, as expected, since this was not a test where we set the strain at a chosen value. Three regions, the linear stage, yielding stage, and densification stage, can be observed in the curves, in agreement with other flexible porous materials [53,54].

At the first stage, with the strain ranging from 0% to ca. 6%, known as the linear stage, where a constant slope of the curve is seen, the main bearing support is ensured by the open pores and the elastic bending. The second stage, the yielding stage (6–50% strain for all samples, except for aerogel_UK with 6–70% strain and XPS with 6–90%), the sample solid skeleton disperses the external force and transfers it to the whole sample, thus avoiding the collapse of the structure.

In the final part, the densification stage, the slope of the stress-strain curve significantly increases due to the densification of the porous structure and the correspondent increase in stiffness of the compressed specimen. In general, a high recovery was obtained (up to 75%) after performing 25% strain compression tests, Figure 4b. After 24 h, the samples recovered to the initial dimensions, observed by the height measurement, except for the XPS. The material’s capacity for recovery to its original shape and the flexibility are important for building applications.

The Young’s modulus was evaluated from the linear region of the stress-strain curve and the values are presented in Table 2. It was observed that the values of Young’s modulus were much smaller for the aerogel_UK and new aerogel_rubber composite, compared with the rubber, rubber_cork composite, or XPS showing the great flexibility of the first. Aerogel-based materials have very high porosity and are fibre-reinforced, thus, they show very high flexibility.

The results indicate very good behaviour for the new aerogel_rubber composite developed by the authors with regard to both flexibility and recovery. This material is also stiffer than the commercial aerogel_UK, even without plastic confinement.

### 3.4. Ageing Effect in the Materials Properties

Table 3 shows the bulk density and thermal conductivity for the new aerogel_rubber composite panels 215 × 215 × 16 mm^3^.

**Figure 5:**
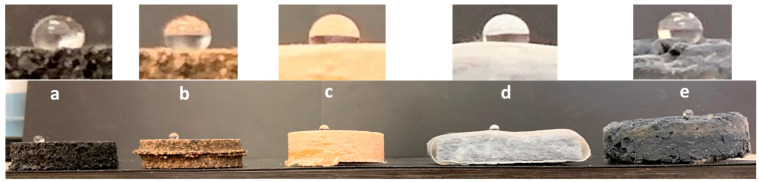
Water droplet images on the surfaces of the different materials, from left to right: (**a**) recycled rubber, (**b**) rubber_cork composite, (**c**) XPS, (**d**) aerogel_UK, and (**e**) new aerogel_rubber composite.

The values shown in the table allow us to conclude that there were no significant changes, in terms of thermal conductivity and density, after the accelerated ageing cycles of the samples. The observed decrease is within the standard deviation interval. The integrity of the panels was kept intact, which contributes to the maintenance of an exceptional insulation performance. Two main factors contributed to this. First, when fibres are added into the aerogel matrix, they are able to resist lateral capillary stresses developed during the drying procedure and thus they can act as supporting skeletons if some shrinkage or collapse tends to occur [53]. The second factor is associated to the modification of the silica matrix by silylation, which provides a highly hydrophobic material at the end, as shown in Figure 5. This hydrophobicity avoids the entrance of water vapour into the matrix, and favours the maintenance of the aerogel performance [40,53]. Moreover, the presence of a silica matrix with entrapped rubber chains also gives resistance to humidity absorption.

In aerogels, the thermal conductivity of the samples is strongly influenced by the-bulk density [48,55], with most of the relevant superinsulating SiO_2_ aerogels commercially available having densities between 80 and 200 kg m^−3^ [56]. All aerogel_rubber composite samples, with different ageing conditions, have shown densities in this range.

**Figure 6:**
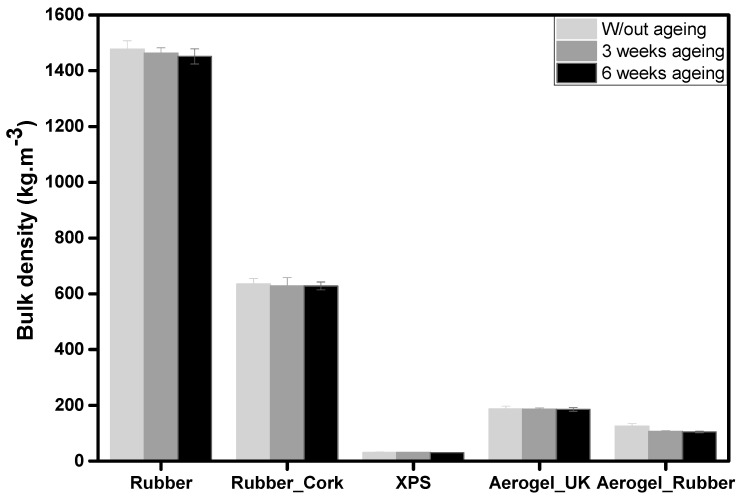
Comparison of measured bulk density for the different materials under different ageing conditions: recycled rubber, rubber_cork composite, XPS, aerogel_UK, and new aerogel_rubber composite.

For the small cylindrical samples (diameter of 40 mm and height of 10 mm) of the new aerogel_rubber composite, differences in ageing can already be seen, as shown in Figure 6. The bulk density was 125.2 ± 8.9 kg·m^−3^ without ageing and 106.4 ± 2.6 kg·m^−3^ and 104.2 ± 3.1 kg·m^−3^ for 3 weeks ageing and 6 weeks ageing, respectively. This decrease in the bulk density may be related to the release of some residual solvents from the synthesis during the heat stages of the ageing. For the other materials (rubber, rubber_cork composite, XPS, and aerogel_UK), the bulk density values did not change significantly and the values were within the measurement error.

**Figure 7:**
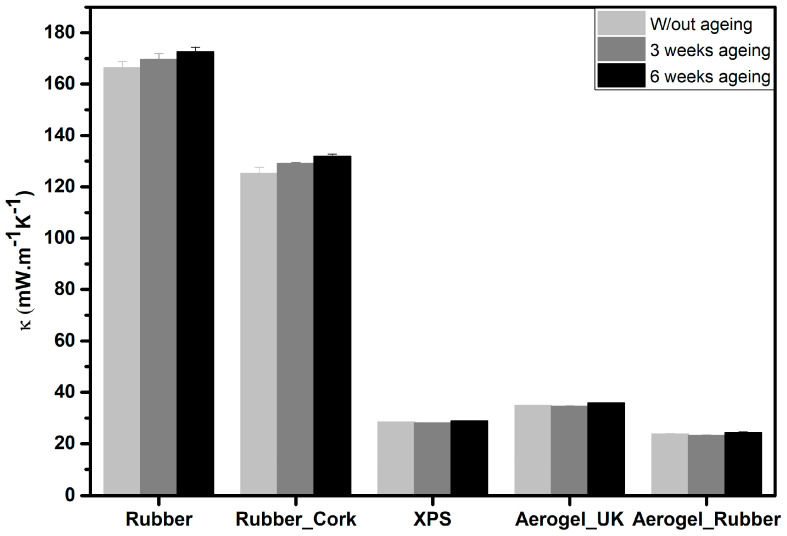
Measured thermal conductivity for the different materials under different ageing conditions: recycled rubber, rubber_cork composite, XPS, aerogel_UK, and new aerogel_rubber composite.

For samples with higher thermal conductivities, ageing has a greater effect on the increase in this property when compared to the more insulating samples, i.e., for recycled rubber, the thermal conductivity increased from 166.3 ± 2.4 mW·m^−1^K^−1^ (without ageing) to 172.5 ± 2.4 mW·m^−1^K^−1^ (6 weeks ageing); for the rubber_cork composite the increase was from 125.3 ± 2.3 mW·m^−1^K^−1^ (without ageing) to 131.9 ± 1.6 mW·m^−1^K^−1^ (6 weeks ageing); in the case of XPS the value changes from 28.5 ± 0.0 mW·m^−1^K^−1^ (without ageing) to 28.9 ± 0.1 mW·m^−1^K^−1^ (6 weeks ageing); for the aerogel_UK the increase was from 34.8 ± 0.1 mW·m^−1^K^−1^ (without ageing) to 35.9 ± 0.5 mW·m^−1^K^−1^ (6 weeks ageing); and for the new aerogel_rubber composite the increase in thermal conductivity was from 23.8 ± 0.2 mW·m^−1^K^−1^ (without ageing) to 24.3 ± 0.7 mW·m^−1^K^−1^ (6 weeks ageing), as shown Figure 7. The greatest difference for thermal conductivity occurred for recycled rubber. In general, the conductivity increased with the number of ageing cycles (humidity and temperature). The here tested materials are mostly hydrophobic; however, with ageing it can be expected that some water vapour remains in the macropores, and this leads to an increase in the thermal conductivity of the materials.

After measuring the thermal conductivity, the samples were placed in an oven at 50 °C for 48 h. It was verified that, after this conditioning, the thermal conductivity of the samples decreased until the values were close to the initial value. The greatest effect on thermal conductivity is, therefore, well explained by the moisture adsorption during the ageing cycles.

The stiffness and the materials’ capacity for recover from compressive stress were also measured and compared without and with 3 and 6 weeks of ageing.

**Figure 8:**
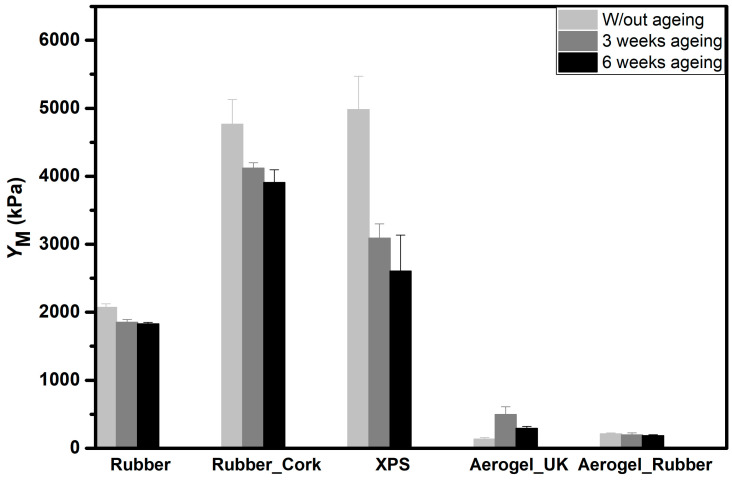
Measured Young’s modulus for the different materials under different ageing conditions: recycled rubber, rubber_cork composite, XPS, aerogel_UK, and new aerogel_rubber composite.

As illustrated in Figure 8, the Young’s modulus (*Y*_M_) decreases as the ageing time increases, leading to a gradual decrease in the stiffness of the materials. This happened for all materials, except for the aerogel_UK samples, probably due to the protective plastic layer, which affects the results for this type of sample. For the other materials, it is anticipated that each chain/layer will have water molecules in its vicinity, which reduces the matrix interconnectivity and increases the chains/layers mobility [57]. This is less pronounced in the aerogel_rubber composite due to its very high hydrophobicity.

**Figure 9:**
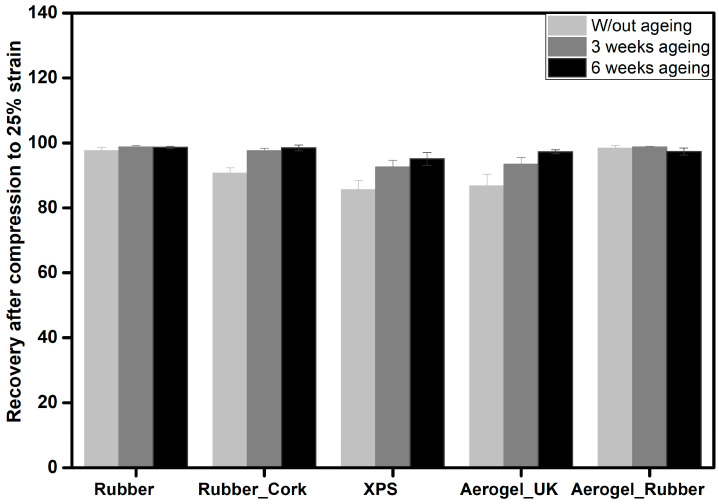
Height recovery of the samples after 25% of compression for the different materials, with different ageing conditions: recycled rubber, rubber_cork composite, XPS, aerogel_UK, and new aerogel_rubber composite.

To further evaluate the capacity of the materials to withstand repeated loads, axial cyclic compression tests were performed until a strain of 25%, followed by recovery, being the obtained results displayed in Figure 9. After the test, the samples only display small reductions in their initial height. After 24 h, the samples were able to completely recover their original size, except the XPS samples, which showed permanent deformations. These results indicate an excellent mechanical performance for the different thermal insulation materials in terms of flexibility, especially for the new aerogel_rubber composites.

The number of ageing cycles led to a progressive increase in the recovery. This is in agreement with the decrease in the Young’s modulus in the same conditions, as the samples become progressively more flexible. Therefore, it can be considered that these different insulation materials can be used in regions with more severe climates without major limitations on their performance.

**Figure 10:**
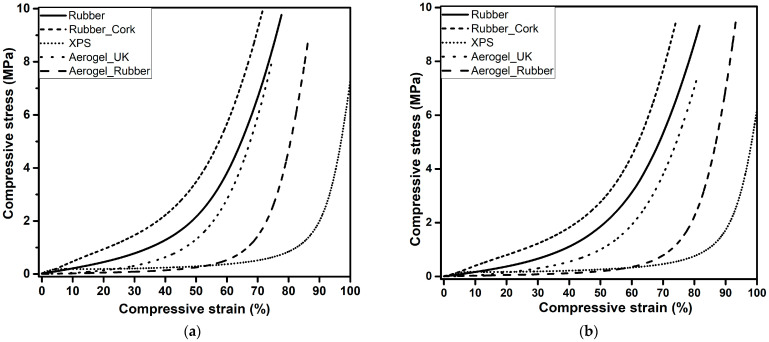
Uniaxial compression test up to densification with a load cell of 3 kN of the rubber, rubber_cork composite, XPS, aerogel_UK, and new aerogel_rubber composite: (**a**) without ageing and (**b)** 6 weeks ageing.

The samples were also submitted to a destructive test, with the load cell of 3 kN, up to the maximum allowed force of the equipment. Figure 10 presents the stress-strain curves obtained from this test, in which the compression progress of the samples contained three stages, as already described in Section 3.3. In general, after ageing, the materials had behaviours very similar to that presented without ageing.

## 4. Conclusions

The thermomechanical behaviour of different insulation materials containing recycled rubber and two others for comparison, namely recycled rubber, rubber_cork composite, XPS, aerogel_UK, and a new aerogel_rubber composite, has been successfully compared for the first time when subjected to the following accelerated ageing conditions—dry-heat, humid-heat, and cold conditions cycle during 3 and 6 weeks. Moreover, the performance, after ageing, of a new super-insulating eco-friendly composite material made from recycled rubber and silica aerogel, recently developed by the authors, was evaluated.

The material with the lowest value of thermal conductivity was the new aerogel_rubber composite (23.8 ± 0.2 mW·m^−1^K^−1^) prepared by the authors, and this was also the one that had the smallest change in the value of thermal conductivity after 3 and 6 weeks of ageing. The greatest difference for thermal conductivity occurred for recycled rubber, which led to an increase from 166.3 ± 2.4 mW·m^−1^K^−1^ (without ageing) to 172.5 ± 2.4 mW·m^−1^K^−1^ (6 weeks ageing), i.e., an increase of 3.6%.

The Young´s modulus (*Y_M_*) decreases as the ageing time increases, leading to a gradual decrease in the stiffness of the materials. This was anticipated to be due to the higher separation of the polymer chains under ageing. The number of ageing cycles led to a progressive increase in the recovery, in agreement with the progressively more flexible behaviour. The samples showed that they practically recovered the initial dimensions when subjected to a cyclic compression until a strain of 25%, except for the XPS that retained a major plastic deformation. The flexibility and the material’s capacity of recovery to its original shape are essential for building applications.

The insulating materials used in this work have a high potential to be applied in buildings with extreme environments, such as those found in the polar regions, very hot climates, and very humid regions. The new aerogel_rubber composites show the highest stability in terms of thermomechanical properties when compared to the insulation materials tested in this work. One limitation of this research is that only two accelerated ageing durations were performed (3 and 6 weeks). Moreover, besides the new superinsulation composite material, only four other commercial insulation materials were compared. As future work, more insulation materials could be tested and the accelerated ageing process duration could be increased to reproduce long-term materials performance degradation under ageing.

## Figures and Tables

**Table 1:** Number of samples prepared for the different thermomechanical tests.

	Aerogel_Rubber Composite	Recycled Rubber	Rubber_Cork Composite ^1^	XPS	Aerogel_UK	Total per Test
Thermal conductivity tests (Hot Disk)	12	16	16	16	16	76
Compression tests	10	10	10	10	10	50
Thermal conductivity tests (HFM)	2	---	---	---	---	2
					Total	128

^1^ Rubber_cork samples were only 5 mm thick and, therefore, 2 pieces were stacked for each sample.

**Table 2:** Measured mechanical properties of the assessed samples.

Samples	Young’s Modulus (kPa)	Compressive Stress after 25% Strain (kPa)	Recovery after Compression to 25% Strain (%)	Maximum Compressive Stress (kPa)	Maximum Strain (%)
Rubber	2099 ± 61	594	97.6 ± 1.0	9423.0	77.5
Rubber_Cork	4373 ± 68	1061	90.6 ± 1.8	9320.0	71.5
XPS	4773 ± 386	185.8	86.7 ± 3.7	9834	100
Aerogel_UK	202 ± 24	128.1	85.6 ± 2.9	9371.5	71.34
Aerogel_Rubber	219 ± 15	72.7	98.3 ± 0.9	9264.2	85.9

**Table 3:** Characterization of the aerogel_rubber composite panels 215 × 215 × 16 mm^2^ with the Heat Flow meter at 23 °C, before and after the ageing tests.

Property	Without Ageing	After 3 Weeks Ageing	After 6 Weeks Ageing
Bulk density (kg·m^−3^)	104.9 ± 2.0	104.3	103.1
Thermal conductivity (mW·m^−1^·K^−1^)	16.4 ± 1.0	16.6 ± 1.0	16.9 ± 1.0
Thermal resistance (m^2^ °C·W^−1^)	0.99	0.96 ± 0.06	0.92 ± 0.05

## Data Availability

Data are contained within the article. The raw/processed data required to reproduce these findings cannot be shared at this time due to technical or time limitations but will be sent upon request.
